# A Knowledge Generation Model via the Hypernetwork

**DOI:** 10.1371/journal.pone.0089746

**Published:** 2014-03-13

**Authors:** Jian-Guo Liu, Guang-Yong Yang, Zhao-Long Hu

**Affiliations:** Research Center of Complex Systems Science, University of Shanghai for Science and Technology, Shanghai, People's Republic of China; University of Adelaide, Australia

## Abstract

The influence of the statistical properties of the network on the knowledge diffusion has been extensively studied. However, the structure evolution and the knowledge generation processes are always integrated simultaneously. By introducing the Cobb-Douglas production function and treating the knowledge growth as a cooperative production of knowledge, in this paper, we present two knowledge-generation dynamic evolving models based on different evolving mechanisms. The first model, named “HDPH model,” adopts the hyperedge growth and the hyperdegree preferential attachment mechanisms. The second model, named “KSPH model,” adopts the hyperedge growth and the knowledge stock preferential attachment mechanisms. We investigate the effect of the parameters 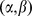 on the total knowledge stock of the two models. The hyperdegree distribution of the HDPH model can be theoretically analyzed by the mean-field theory. The analytic result indicates that the hyperdegree distribution of the HDPH model obeys the power-law distribution and the exponent is 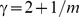. Furthermore, we present the distributions of the knowledge stock for different parameters 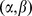. The findings indicate that our proposed models could be helpful for deeply understanding the scientific research cooperation.

## Introduction

Network science provides an useful perspective for the study of knowledge diffusion [1], [2]. Accompanying with the increasing popularity of the complex network researches, many scholars concentrate on exploring how knowledge diffuses on the fixed network topology structures. Cowan and Jonard [3] compared knowledge diffusion in a range of network structures, from the regular network to the fully-random network. Kim and Park [4] also measured the knowledge diffusion in regular, random and small-world networks by using a model that integrated the knowledge creation and the knowledge exchange; and a similar conclusion as Cowan and Jonard's was drawn that the small-world network was the most efficient structure to achieve the knowledge diffusion. Besides the small-world property, the scale-free property is another topological structure. Correspondingly, diffusion in scale-free networks has also been widely-discussed [5]–[8]. Tang *et.al* [6], [7] argued that scale-free structure was more effective for knowledge transfer. The advantage of the scale-free network for knowledge diffusion had also been shown in Lin and Li's work [8], which provided a numerical test of knowledge diffusion in regular, random, small-world, and scale-free networks. In addition, Xuan *et.al* [9] adopted the agent-based modeling approach to compare the performance of knowledge transfer in a series of networks which differed from one another in their “knowledge-connection” structures. These researches could be helpful for understanding how the network properties influence the performance of knowledge diffusion, but they ignored the evolution of the network. Therefore, that knowledge diffuses on the dynamic evolving networks has recently caught great attentions [10], [11]. Morone and Taylor [10]considered that the network structure would be affected by individual behaviors and interaction, and investigated knowledge diffusion dynamics and the evolution and formation of the network in the process of interactive learning. Guimerà et.al [11] proposed a model for the self-assembly of creative teams, and found that the emergence of a large connected community of practitioners could be described as a phase transition. Team assembly mechanisms determined both the structure of the collaboration network and team performance for teams derived from both artistic and scientific fields.

The aforementioned efforts contribute noticeably for improving the understanding of knowledge-diffusion in all sorts of social networks. However, in the scientific collaboration network, a new node collaborates with the old nodes to co-author a paper. That the new node joins the network is a process of network construction. Co-authoring a paper is a process of knowledge generation. Therefore, the network construction process and the knowledge generation process can be integrated simultaneously. Furthermore, people tend to create and diffuse knowledge by coauthoring papers in the scientific collaboration systems. In complex network, an edge relates only a pair of nodes. This research technique of scientific collaboration network can not express the information of papers from the viewpoint of complex network, where the nodes represent the authors and the edges indicate the cooperative relationship between them [12]–[14]. In this paper, we argue that the hypernetwork is more feasible to analyze the knowledge diffusion in the scientific collaboration system. In the hypernetwork, a hyperedge can contain more than two nodes. Thus, it is useful to represent the collaboration network as a hypernetwork in which nodes represent authors and hyperedges represent papers that have been coauthored by the groups of authors [15]–[17]. By considering the collaborative scientific behaviors, Hu *et.al* [18] proposed a model for evolving hypernetwork based on the hypergraph theory. Furthermore, the scientific research activity engaged in the scientific collaboration networks is not only the process of knowledge dissemination, but also the process of knowledge generation.

Inspired by the above ideas, we present two knowledge-generation dynamic evolving models among the scientific collaboration hypernetwork by integrating the hypernetwork structure evolution and knowledge generation processes simultaneously. By introducing the Cobb-Douglas production function [19] to the knowledge generation process, the two models treat the knowledge growth caused by the scientific research cooperation as a cooperative production of knowledge products. The first model named “HDPH model” adopts the hyperedge growth and the hyperdegree preferential attachment mechanisms. And the created knowledge stock is equally divided by contributors. The second model named “KSPH model” adopts the hyperedge growth and the knowledge stock preferential attachment mechanisms. And the contributor's knowledge increases by an amount proportional to the contributor's owned knowledge stock. We examine the effect of the different parameters 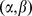 of the production function on knowledge generation, and analyze the distribution of knowledge stock. The results indicate that our model to some extent, may reflect the scientific research cooperation situation.

The remainder of our paper is organized as follows. In Sec. II, the knowledge-generation dynamic evolving models are given. In Sec. III, we investigate the process of knowledge generation, examine the effect of the different parameters 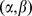 on knowledge generation and analyze the knowledge stock distribution and hyperdegree distribution. The conclusions and discussions are given in Sec. IV.

## The Models

Scientific research collaboration is a process of absorbing each other's knowledge and co-creating new knowledge. In economics, the Cobb-Douglas functional form of production functions is widely used to represent the relationship of an output to inputs, particularly physical capital and labor, and the amount of output that can be produced by those inputs [19]. The scientific research output can be represented by published papers. The output of papers needs to invest manpower and knowledge. Therefore, we introduce the Cobb-Douglas production function to the knowledge generation process. The scientific knowledge generation is that the scientific researchers create new knowledge on the basis of the original knowledge accumulation. We assume that the created knowledge stock of a paper depends on the co-authors' knowledge level as well as the number of the co-authors. The knowledge production function can be defined as 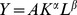, where 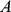 denotes the comprehensive creative level, 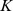 denotes the average knowledge stock of coauthors, 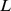 is the number of coauthors, and 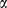, 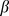 is the corresponding elasticity coefficient. Furthermore, the local world effect of the hypernetwork is introduced.

In the literatures on knowledge transfer models, knowledge has been represented in several ways, e.g., by a stock [10], by a vector of real positive scalars [3], by a pair constituting a scalar and an angle [20], and by a “tree” of activated nodes [21]. In our model, the stock representation suggested by Morone and Taylor is adopted [10].

Let 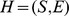 be a simple and finite hypernetwork with the node set 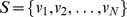 and the hyperedge set 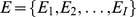, where 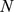 is the node number, 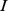 is the hyperedge number, 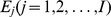 is a nonvoid subset of 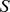. Let 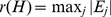, 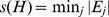. If 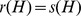, we say that 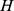 is a uniform hypernetwork; Otherwise 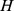 is a non-uniform hypernetwork [22], [23]. The definition of hyperdegree for a vertex in a hypergraph is simply the number of hyperedges attached to that vertex [24], [25]. In this paper, the hyperdegree for a node in a hypernetwork is defined as the number of the hyperedge attached to that node.

### HDPH model

The HDPH model adopts the hyperedge growth and the hyperdegree preferential attachment mechanisms. The created knowledge stock is equally divided by contributors. The knowledge generation is based on the growth process of the hypernetwork. The HDPH model could be constructed in the following way:

- Initial condition: The hypernetwork consists of 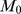 nodes and 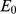 hyperedges in the initial stage. Each node holds some “knowledge”, which is defined by Morone and Taylor [10].
- Determination of the local-world: Select 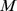
(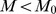) nodes randomly from the existing hypernetwork as the local world at each time step.
- Hyperedge growth: Add a new hyperedge encircling a newly added node and 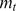 selected nodes in the local world determined in (ii) at time step 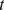, where 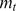 is a value selected randomly from the set 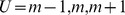 and obeys a uniform distribution, and 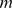 is a preset fixed value and 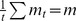. Each newly added node 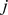
's knowledge stock is initialized by setting 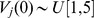.
- Hyperdegree preferential attachment: Choose 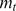 nodes in the local world to construct the new hyperedge 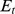, the probability 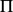 for node 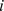 is selected depends on the hyperdegree 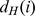 of node 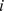, such that
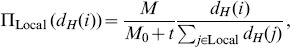
(1)where 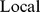 denotes the local world node set and the hyperdegree 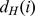 is defined as the number of hyperedges node 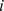 belonging to.
- Knowledge generation: Suppose that the knowledge stock created by the new hyperedge 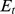 is 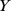, then
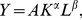
(2)where 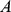 denotes the comprehensive creative level, 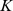 denotes the average knowledge stock of the hyperedge 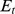
's nodes, and 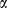, 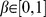 is the corresponding elasticity coefficient. The change of the knowledge status quo of node 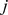 is formulated as follows:
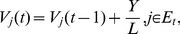
(3)where 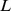 is the number of the hyperedge 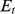
's nodes and 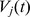 denotes the knowledge status quo of node 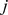 at time 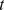.

**Figure 1:**
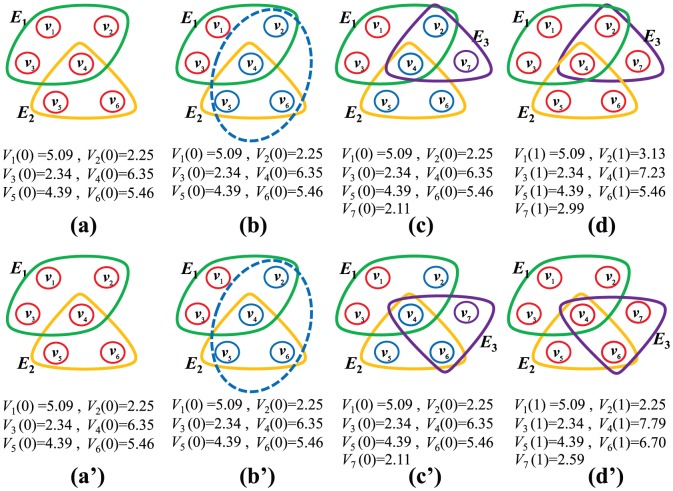
Schematic illustration of the evolving process at each time step of the knowledge-generation hypernetwork models for the case of the number of initial nodes 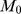 = 6, the local-world size 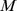 = 4 and the number of selected nodes 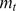 = 2. 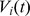
 is defined as the node 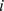
's knowledge stock at time step 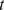. (a) HDPH model: starting from two hyperedges 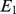, 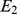 described by closed curves, which contain six nodes with the knowledge stock 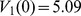, 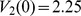, 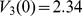, 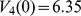, 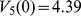, 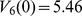, respectively. (b) Select four nodes randomly (shown as four blue hollow circles) from the existing hypernetwork as the local world of a new coming node. All nodes' knowledge stock remain unchanged. (c) A newly added hyperedge 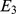 prefers to encircle a new coming node 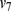 with the initial knowledge stock 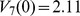 and two existing nodes 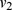, 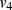 with the more hyperedges in the local-world. All old nodes' knowledge stock remain unchanged. (d) The nodes 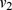, 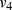, 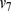 existing in the newly added hyperedge 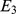 co-author a paper to create new knowledge. The created knowledge stock equals to 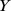, and 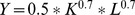, where 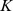 is the average knowledge stock of the nodes 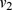, 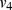 and 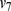; 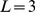 is the number of the hyperedge 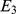
's nodes. The knowledge status quo of node 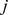 is formulated as follows: 
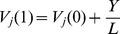
, 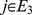. The rest of nodes' knowledge status quo remain unchanged. The HDPH model after one time step is presented. (a′) KSHP model: starting from two hyperedges 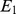, 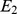 described by closed curves, which contain six nodes with the knowledge stock 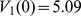, 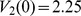, 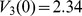, 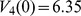, 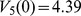, 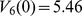, respectively. (b′) Select four nodes randomly (shown as four blue hollow circles) from the existing hypernetwork as the local world of a new coming node. All nodes' knowledge stock remain unchanged. (c′) A newly added hyperedge 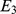 prefers to encircle a new coming node 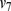 with the initial knowledge stock 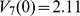 and two existing nodes 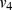, 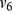 with the more knowledge stock in the local-world. All old nodes' knowledge stock remain unchanged. (d′) The nodes 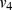, 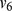, 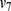 existing in the newly added hyperedge 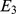 co-author a paper to create new knowledge. The created knowledge stock equals to 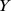, and 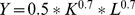, where 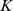 is the average knowledge stock of the nodes 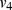, 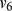 and 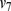; 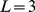 is the number of the hyperedge 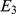
's nodes. The knowledge status quo of node 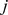 is formulated as follows: 
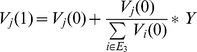
, 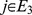. The rest of nodes' knowledge status quo remain unchanged. The KSHP model after one time step is presented.

After 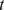 time steps, this model leads to a hypernetwork with 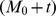 nodes, 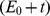 hyperedges. The total knowledge stock 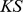 of the hypernetwork is 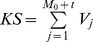. Figure 1 (*a*)∼(*d*) shows the evolving process of the HDPH model.

### KSPH model

The KSPH model adopts the hyperedge growth and the knowledge stock preferential attachment mechanisms. The contributor's knowledge increases by an amount proportional to the contributor's owned knowledge stock. The knowledge generation is based on the growth process of the hypernetwork. Meanwhile, the growth process of the hypernetwork depends on the knowledge stock. The KSPH model could be constructed in the following way:

- Initial condition: The hypernetwork consists of 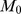 nodes and 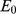 hyperedges in the initial stage. Each node holds some “knowledge”, which is defined by Morone and Taylor [10].
- Determination of the local-world: Select 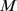
(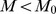) nodes randomly from the existing hypernetwork as the local world at each time step.
- Hyperedge growth: Add a new hyperedge encircling a newly added node and 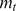 selected nodes in the local world determined in (ii) at time step 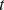, where 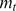 is a value selected randomly from the set 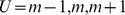 and obeys a uniform distribution, and 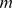 is a preset fixed value and 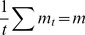. Each newly added node 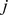
's knowledge stock is initialized by setting 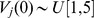.
- Knowledge stock preferential attachment: Choose 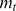 nodes in the local world to construct the new hyperedge 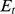, the probability 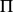 for node 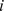 is selected depends on the knowledge stock 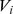 of node 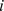, such that
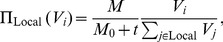
(4)where 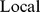 denotes the local world node set.
- Knowledge generation: Suppose that the knowledge stock created by the new hyperedge 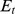 is 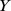, then
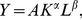
(5)where 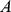 denotes the comprehensive creative level, 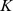 denotes the average knowledge stock of the hyperedge 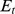
's nodes, and 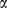, 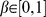 is the corresponding elasticity coefficient. The change of the knowledge status quo of node 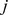 is formulated as follows:
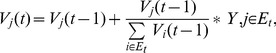
(6)where 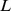 is the number of the hyperedge 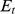
's nodes and 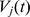 denotes the knowledge status quo of node 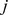 at time 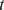.

After 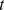 time steps, this model leads to a hypernetwork with 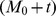 nodes, 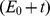 hyperedges. The total knowledge stock 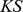 of the hypernetwork is 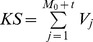. Figure 1 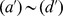 shows the evolving process of the KSPH model.

## Numerical Simulation

The evolving process of the above two models may be divided into two stages. The first stage generates an initial hypernetwork for local-world evolving process, which contains 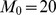 nodes and 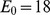 hyperedges. This initial hypernetwork adopts the hyperedge global preferential attachment mechanism. In the second stage, the hypernetwork model starts with 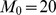 nodes and 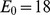 hyperedges. At each time step 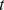, a new hyperedge is added to the system and will encircle a new coming node and 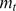 selected nodes in the local world. The parameters are set as follows: the mean values 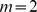, the size of the local world 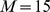, and the hypernetwork size 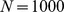. Each node has a knowledge stock, initialized by setting 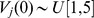. Parameter 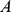, measuring the comprehensive creative level of the knowledge-generation function, is set to 0.5.

### The total knowledge stock

**Figure 2:**
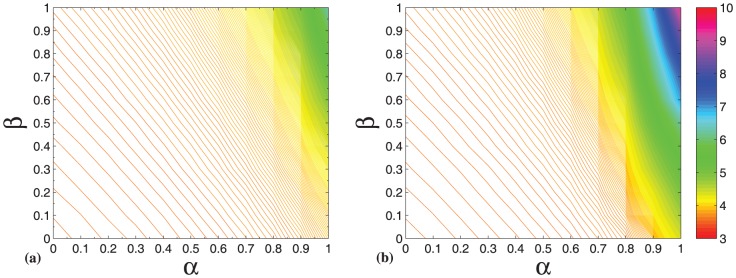
The logarithmic values log 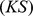 of the total knowledge stock 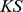, 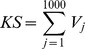, as a function of different parameter combinations 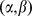 on the contour map. The number of nodes 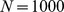. 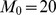 and 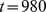. Each simulation result is obtained by averaging over 100 independent runs. (a) HDPH model. (b) KSPH model.

In order to investigate the effect of the parameters 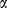 and 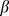 on the total knowledge stock of the two models, we independently conduct 121 groups of experiments, respectively. The parameters 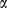 and 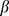 are set as the elements of the set 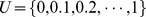, respectively. Figure 2 shows the total knowledge level of the two models under different parameter combinations 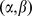. The findings indicate that the total knowledge stock of the two models will both become larger as 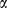 or 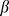 increases. For the same parameters 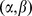, the total knowledge stock of KSPH model is higher than that of HDPH model.

### Knowledge stock distribution

We analyze the knowledge stock distribution of the two models with different parameters 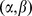. When analyzing the statistical analysis of the knowledge-stock distribution, since with real numbers knowledge levels never become identical, we make the knowledge stock of each node round into integers.

**Figure 3:**
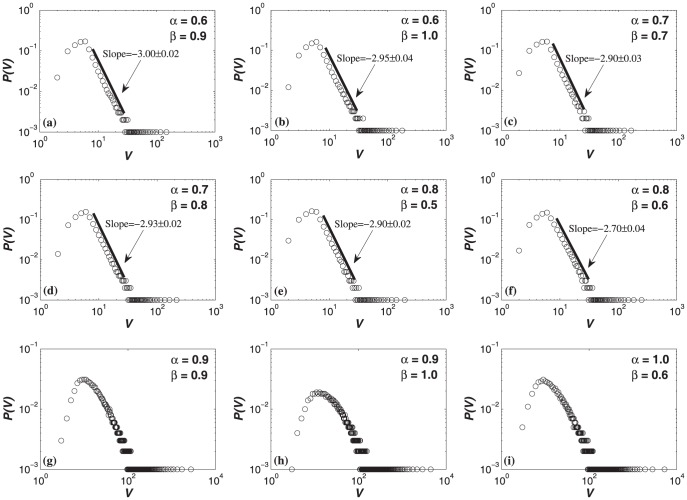
Probability distribution of the knowledge stock 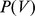 in the HDPH model on a logarithmic scale with the different parameter combinations 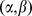. 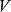
 is defined as the knowledge stock. Not all knowledge-stock distributions of HDPH model for 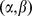 values exhibit a power-law form. 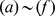 The knowledge stock distribution exhibits a power-law form. 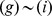 The knowledge stock does not exhibit a power-law form. Each simulation result is obtained by averaging over 100 independent runs.

For the HDPH model, not all knowledge-stock distributions for 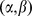 values exhibit a power-law form. Figure 3 displays 9 subgraphs about the probability distribution of the knowledge stock with different parameters 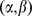. The knowledge stock exhibits a power-law form in Fig. 3(*a*)∼(*f*), while the knowledge stock does not exhibit a power-law form in Fig. 3(*g*)∼(*i*). Therefore, in the knowledge generation hypernetwork generated by the evolution mechanism of HDPH model, the knowledge stock distribution has a variety of forms.

**Figure 4:**
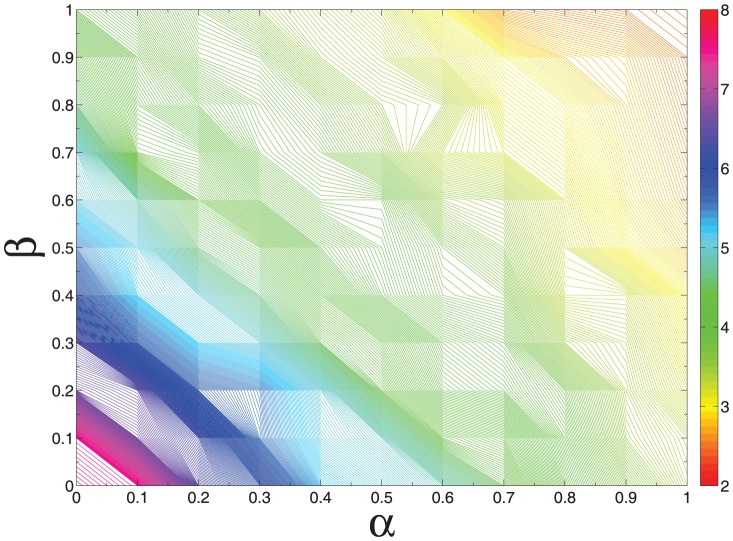
The power-law exponent of the knowledge stock distribution in the KSPH model as a function of different parameters 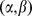 on the contour map. All knowledge-stock distributions of KSPH model for 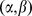 values exhibit a power-law form. Each simulation result is obtained by averaging over 100 independent runs.

For the KSPH model, all knowledge-stock distributions for 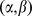 values exhibit a power-law form. Figure 4 displays the power exponents of knowledge-stock distributions with different 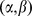 values. When 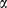 remains unchanged, the power exponent of knowledge-stock distributions gradually decreases as 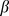 increases. Similarly, when 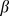 remains unchanged, the power exponent of knowledge-stock distributions also gradually decreases as 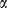 increases. Therefore, in the knowledge generation hypernetwork generated by the evolution mechanism of KSPH model, the knowledge stock distributions all exhibit a power-law form.

### Hyperdegree analysis

The hyperdegree distribution of HDPH model can be theoretically analysed by the mean-field theory. The analytic result indicates that the hyperdegree distribution is independent of the local-world size 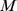 and exhibits a pow-law distribution, i.e., 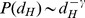, where the exponent 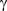 is correlated with the mean value 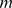, 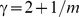 (see File S1). Zlatić *et.al* [25]defined and analyzed the statistical properties of tripartite hypergraphs. The results showed that the hyperdegree distributions for users, tags, and resources also obeyed the power law distribution. Although the probability distribution of the knowledge stock with some parameter combinations 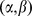 conforms to a power law distribution, it is independent of the hyperdegree distribution of HDPH model. Because in the same hypernetwork topology structure, the probability distribution of the knowledge stock with the different parameter combinations 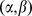 is different. And not all knowledge-stock distributions for 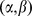 values exhibit a power-law form.

**Figure 5:**
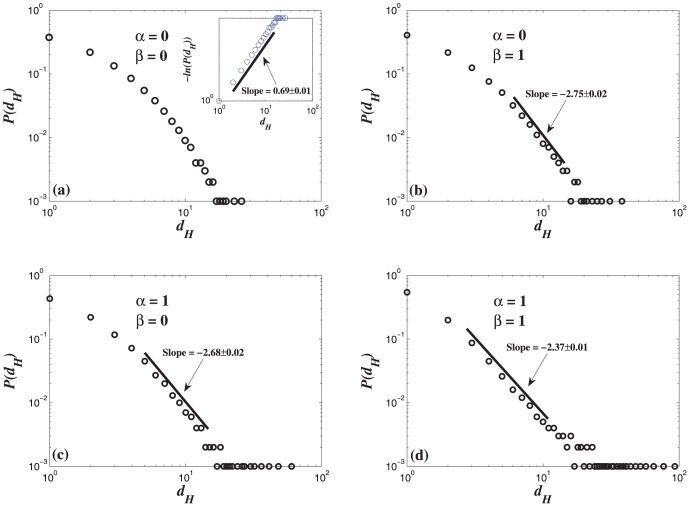
Probability distributions of the hyperdegree 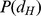 in the KSPH model on a logarithmic scale with the different parameters 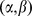. The node number 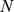 = 1000, the size of local-world 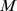 = 15, and the mean value 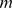 = 2. (a) When 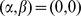, the hyperdegree distribution obeys the stretched exponential distribution, as 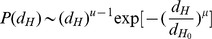, where 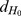 is a constant and 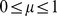 is the characteristic exponent. If 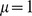, it is a normal exponential distribution. Using log(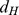) as *x*-axis and log(−log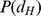) as *y*-axis, if the corresponding curve can be well fitted by a straight line, then the slope equals 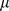. In the inset, we plot the linear correlation between log(−log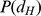) and log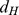. (b) When 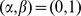, for big values of 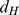, the hyperdegree distribution follows a power-law distribution and the exponent is approximately equal to 2.75. (c) When 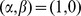, for big values of 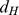, the hyperdegree distribution follows a power-law distribution and the exponent is approximately equal to 2.68. (d) When 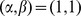, the hyperdegree distribution follows a power-law distribution and the exponent is approximately equal to 2.37. Each simulation result is obtained by averaging over 100 independent runs.

**Figure 6:**
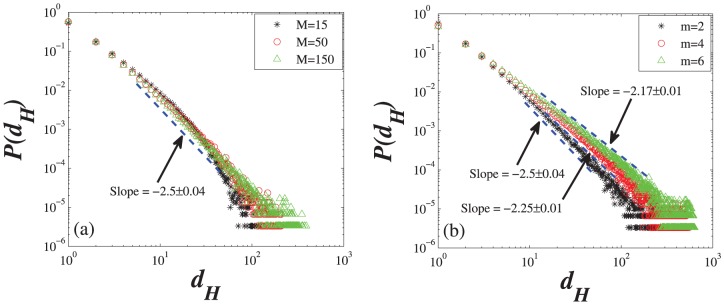
Probability distributions of the hyperdegree 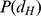 in the HDPH model on a logarithmic scale with the different parameters. (a) When 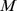 = 15, 50, and 150, the node number and the mean value 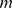 are set as 10000 and 2, respectively. The hyperdegree distribution is approximately independent of the local-world size 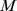, and the power-law exponent is approximately equal to 2.5. (b) When 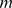 = 2, 4, and 6, the node number and the value 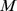 are set as is 10000 and 150, respectively. The power-law exponent of hyperdegree distributions decrease as 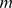 increases, which are approximately equal to 2.5, 2.25, 2.17 in three cases of 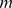 = 2, 4, and 6, respectively. Each simulation result is obtained by averaging over 30 independent runs.

The KSPH model considers that the evolution of the hypernetwork structure will be affected by the knowledge stock. The growth process of the hypernetwork depends on the knowledge stock. Therefore, the hyperdegree distribution of KSPH model is difficult to be theoretically analyzed. We numerically investigate the hyperdegree distribution 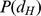 of KSPH model with 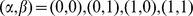 in Fig. 5. The hyperdegree distribution 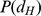 of the HDPH model is shown in Fig. 6. When 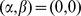, the hyperdegree distribution 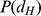 follows a stretched exponential distribution with exponent 0.69. When 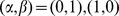, for big values of 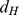, the hyperdegree distribution 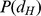 displays a power-law behavior and the exponent is approximately equal to 2.75 and 2.68. When 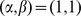, the hyperdegree distribution 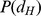 follows a power-law distribution with exponent 2.37.

The numerical simulations of the hyperdegree distribution 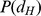 is given in Fig. 6. Figure 6(a) shows the hyperdegree distribution 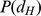 with the mean value 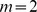 and different local-world sizes 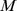. Figure 6(b) shows the case of the same local-world size 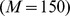 and different mean values 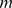. As seen from Fig. 6(a), the hyperdegree distribution 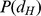 of HDPH model closely overlap together as 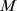 increases. Figure 6(b) shows that the power-law exponent 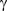 of hyperdegree distribution decrease as 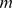 increases. The simulation results are quite consistent with the theoretical ones. We can easily find that the hyperdegree distribution of HDPH model is approximately independent of the local-world size 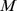 and exhibits a pow-law distribution, i.e., 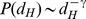, where the exponent 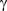 is correlated with the mean value 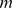 (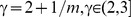), and the power exponent of the empirical results of the social tagging hypernetwork, 2.28 and 2.13, is also in (2,3].

## Conclusions and Discussions

### Summary

The knowledge generation and diffusion in the networks often comes with the network structure evolution. By integrating the hypernetwork structure evolution and knowledge generation processes together, we present two knowledge-generation dynamic evolving hypernetwork models (HDPH model and KSPH model). The two models are not based on a static perspective as was the configuration model, but on a dynamical mechanism to construct the hypernetworks. The HDPH model adopts the hyperedge growth and the hyperdegree preferential attachment mechanisms. The created knowledge stock is equally divided by contributors. The KSPH model adopts the hyperedge growth and the knowledge stock preferential attachment mechanisms. The contributor's knowledge increases by an amount proportional to the contributor's owned knowledge stock. Furthermore, the knowledge generation process is simultaneous with the hypernetwork structure evolution.

We investigate the effect of the parameters 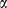, 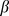 on the total knowledge stock of the two models. The experimental results indicate that the total knowledge stock of the two models will both become larger as 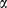 or 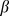 increase. For the same parameters 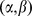, the total knowledge stock of KSPH model is higher than that of HDPH model.

In addition, we also analyze the knowledge stock distribution of the two models with different parameters 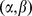. For the HDPH model, not all knowledge-stock distributions for 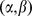 values exhibit a power-law form. While for the KSPH model, all knowledge-stock distributions for 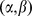 values exhibit a power-law form. When 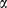 remains unchanged, the power exponent of knowledge-stock distributions gradually decreases as 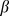 increases. Similarly, when 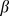 remains unchanged, the power exponent of knowledge-stock distributions also gradually decreases as 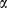 increases. Therefore, in the knowledge generation hypernetwork generated by the evolution mechanism of HDPH model, the knowledge stock distribution has a variety of forms. While in the knowledge generation hypernetwork generated by the evolution mechanism of KSPH model, the knowledge stock distributions all exhibit a power-law form.

The hyperdegree distribution of HDPH model can be theoretically analysed by the mean-field theory. The hyperdegree distribution of HDPH model follows a power-law distribution with exponent 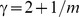. While for the KSPH model, the growth process of the hypernetwork depends on the knowledge stock. Therefore, the hyperdegree distribution of KSPH model is difficult to be theoretically analyzed. However, the simulation results show that the hyperdegree distribution of KSPH model follows the stretched exponential distribution and power-law distribution.

### Limitations and future work

Firstly, in this work, we introduce the Cobb-Douglas production function in economics to the knowledge generation process. In economics, the Cobb-Douglas production function is applicable to industrial products. We try to use the C–D function to model the knowledge production. Whether it is applicable or not, the Cobb-Douglas form in the knowledge production needs to be tested against statistical evidence in the future work.

Secondly, in our models, the knowledge stock's increase of each author is from the overall achievement in a team-based knowledge production process. We propose two knowledge growth mechanisms. One is the created knowledge stock being equally divided by contributors, and the other one is the contributor's knowledge increasing by an amount proportional to the contributor's owned knowledge stock. However, knowledge is different from physical matters. In the real scientific collaboration activities, how to measure the increased knowledge stock of each author is a very complicated problem. Therefore, as another direction of the future work, we need to design a more reasonable knowledge growth mechanism to cater for complicated situation.

Thirdly, in this paper, the knowledge has been represented by a stock. At the same time, how to measure the knowledge stock of a paper in the real life is an open question. And when applying the empirical data of the real scientific collaboration hypernetwork to statistically analyse the process of knowledge generation and dissemination, we must address the problem. In the future work, it is a worthwhile problem to make empirical analysis on the knowledge generation and dissemination process in the scientific collaboration hypernetwork.

Last but not least, we in this paper have proposed two knowledge-generation dynamic evolving hypernetwork models and studied the knowledge generation process. But the corresponding empirical research about knowledge generation among the scientific collaboration hypernetwork is generally absent. As a complement to this work, we model the real knowledge generation and dissemination cases and statistically analyze the knowledge stock characteristics. Due to the intrinsic intractability of knowledge and knowledge generation, such empirical study is essentially challenging, but this issue is worthy of inquiry in the context of scientific collaboration.

## Supporting Information

File S1 (PDF)
