# Supplementary material for: A Knowledge Generation Model via the Hypernetwork
Source: PLoS One. 2014 Mar 13;9(3):e89746. doi: 10.1371/journal.pone.0089746 (PMC3953075; doi:10.1371/journal.pone.0089746)
Supplement: File S1 — (PDF) [file pone.0089746.s001.pdf]

Supporting Information

The theoretical analysis and numerical simulation of the hyperdegree in the HDPH model have been presented in our previous work [17].

To investigate the effect of the local-world property and different mean values m on the hyperdegree of HDPH model, we explore two groups of experiments. The first one is to investigate the effect of the local-world size M on the evolving results with the initial conditions of $M_0 = 160$, $E_0 = 158$, $m = 2$. The second one is to explore the effect of the mean values m on the evolving results. Initially, we set $M_0 = 160$, $E_0 = 158$, and $M = 150$. The size of local world M is obtained according to the theory of Dunbar's number that the cognitive limit number of social relationships we can maintain is around 150 [?, ?].

Theoretical analysis of the hyperdegree

The hyperdegree $d_H(i)$ is defined as the number of the hyperedges node i belonging to. For instance, in scientific collaboration hypernetworks, the hyperdegree of node i is the number of papers that the author i wrote. The hyperdegree distribution $P(d_H)$ is defined as the probability that one randomly selected node possesses d_H hyperedges, which is a very important quantity to characterize the hypernetwork structure. The theoretical results are given based on the mean-field approach.

The generation process of the HDPH model may be divided into two stages. The first stage generates an initial hypernetwork for local-world evolving process, which contains M_0 nodes and E_0 hyperedges. In the second stage, the HDPH model starts with M_0 nodes and E_0 hyperedges. At each time step t, a new hyperedge is added into the system and will encircle a new coming node and m_t selected nodes in the local world. At time step t, the growing hypernetwork consists of $(M_0 + t)$ nodes enclosed by $(E_0 + t)$ hyperedges. When choosing m_t nodes from the local world, the probability that the node i is selected could be given as follows

$$m[\prod_{\text{Local}} d_H(i)][1 - \prod_{\text{Local}} d_H(i)]^{m-1} \approx m \prod_{\text{Local}} d_H(i), \tag{1}$$

where $\frac{1}{t}\sum m_t = m$, and Local, $d_H(i)$ denote the local world node set and the hyperdegree of node i, respectively. Let $d_H(t)$ be the hyperdegree of node i at time t. Consequently, $d_H(t)$ satisfies the dynamical equation

$$\frac{\partial d_H}{\partial t} \approx m \prod_{\text{Local}}(d_H(i)) = \frac{mM}{M_0+t} \frac{d_H(i)}{\sum_{j\in\text{Local}} d_H(j)}. \tag{2}$$

Because the random selection of the M nodes contributes to a local world connection at each time step t, the cumulative degree of the local world depends on the random selection. In general, to simplify the following analysis, we assume that

$$\sum_{j\in\text{Local}} d_H(j) = \langle d_H(i)\rangle M, \tag{3}$$

where the average hyperdegree $\langle d_H(i)\rangle = \frac{(D_0+(m+1)t)}{(M_0+t)}$ and D_0 is the sum of all nodes' hyperdegree in the initial hypernetwork generated by the first stage. Substituting Eq. (3) into Eq. (2), which leads to

$$\frac{\partial d_H}{\partial t} = \frac{m}{D_0+(m+1)t} d_H(t). \tag{4}$$

When D_0 is small and t is large, Eq. (4) is approximately equal to

$$\frac{\partial d_H}{\partial t} = \frac{m}{(m+1)t} d_H(t). \tag{5}$$

Suppose that node i is added into the hypernetwork at time t_i. Since the initial condition that every node i is $d_H(t_i) = 1$, the solution of the above equation would be

$$d_H(t) = (\frac{t}{t_i})^{\frac{m}{m+1}}. \tag{6}$$

Using Eq. (6), the probability that a node has a hyperdegree $d_H(t)$ smaller than d_H, $P(d_H(t) < d_H)$, can be written as

$$P(d_H(t) < d_H) = P(t_i > \frac{t}{d_H^{(m+1)/m}}). \tag{7}$$

Suppose the nodes are added equally into the hypernetwork at each time step, the t_i values have a constant probability density $\rho(t_i) = \frac{1}{t}$. Substituting it into Eq. (7), one has

$$P(t_i > \frac{t}{d_H^{(m+1)/m}}) = 1 - \frac{t}{d_H^{(m+1)/m} t}. \tag{8}$$

The hyperdegree distribution $P_H(d_H)$ can be obtained by

$$P(d_H) = \frac{\partial P(d_H(t) < d_H)}{\partial d_H} = \frac{(m+1)}{m} d_H^{-(2+\frac{1}{m})}. \tag{9}$$

Thus the probability distribution $P(d_H)$ has a generalized power-law form

$$P(d_H) = \frac{m+1}{m} d_H^{-(2+\frac{1}{m})}, \tag{10}$$

where the power exponent equals to $\gamma = (2 + 1/m)$.
